# Distribution and treatment of clavicular fractures in monotrauma and polytrauma patients

**DOI:** 10.1186/1752-2897-8-17

**Published:** 2014-11-27

**Authors:** Steven Ferree, Jacqueline JEM van Laarhoven, R Marijn Houwert, Falco Hietbrink, Egbert Jan MM Verleisdonk, Luke PH Leenen

**Affiliations:** Department of Surgery, Diakonessenhuis, Utrecht, The Netherlands; Department of Surgery, University Medical Center Utrecht, Utrecht, The Netherlands

## Abstract

**Background:**

Although extensive research for the optimal treatment of clavicle fractures has been performed, comparative studies between monotrauma and polytrauma patients are lacking.

**Objective:**

To compare fracture distribution and treatment in monotrauma and polytrauma patients with a clavicle fracture.

**Methods:**

Single center retrospective cohort study. Fractures were classified by the Robinson classification. Monotrauma patients sustained only a clavicle fracture or a clavicle fracture plus a minor abrasion, hematoma, or superficial skin lesion leading to an Injury Severity Score (ISS) of 4 or 5 respectively. Polytrauma patients had an ISS ≥16 as a result of injury in 2 or more Abbreviated Injury Scale (AIS) regions.

**Results:**

154 monotrauma and 155 polytrauma patients with a clavicle fracture were identified. Monotrauma patients had a higher incidence of Type IIB fractures (displaced midshaft) compared to polytrauma patients (P = 0.002). No difference was observed regarding Type I (medial) and Type III (lateral) fractures. In monotrauma patients, Type IIB fractures were treated operatively more frequently (P = 0.004). The initial treatment for Type I and Type III fractures did not differ between monotrauma and polytrauma patients.

**Conclusions:**

Monotrauma patients had a higher incidence of displaced midshaft clavicle fractures compared to polytrauma patients, and monotrauma patients with displaced midshaft clavicle fractures were treated operatively more frequently. No differences were found in the distribution and treatment of medial and lateral clavicle fractures.

## Introduction

Clavicle fractures account for approximately 5% of all fractures. Eighty percent of clavicle fractures occur in the midshaft and 50% of these fractures are displaced [1, 2]. Lateral (17%) and medial fractures (3%) are less common [1, 2]. In polytrauma patients, incidences of up to 10% are reported for clavicle fractures [1, 2].

The indication for operative treatment of midshaft and lateral clavicle fractures is increasingly being based on fracture classification and displacement [3–5]. A detailed classification system for clavicle fractures is provided by Robinson et al [6]. This classification describes the anatomical location and magnitude of the fracture displacement. Recent studies report low rates of non-union and early return to normal function of operatively treated patients with displaced midshaft and lateral clavicle fractures. However, these studies are based on monotrauma patients [3, 4, 7–9].

Although extensive research for the optimal treatment of clavicle fractures has been performed, comparative studies between monotrauma and polytrauma patients are lacking. The aim of this study was to compare distribution and treatment in monotrauma and polytrauma patients with a clavicle fracture.

## Material and methods

This was a single center retrospective cohort study. Patient data for this study were derived from the Dutch National Trauma Database (DNTD) for the central Netherlands area and electronic patient documentation. The Dutch National Trauma Database contains prospectively collected documentation on sustained injuries in trauma patients. Patients included in this study were selected using the diagnostic code (ICD-code) for a clavicle fracture at the University Medical Center Utrecht, a level 1 trauma center, from January 2007 until December 2011. For all patients, age, gender, trauma mechanism, injured side, choice of treatment, department of admission (surgical ward, medium care unit, intensive care unit, operation room) and discharge facility were collected. Exclusion criteria for this analysis were age <16 years, no imaging studies available, initial treatment at another hospital, no follow-up after Emergency Room visit, and a bilateral clavicle fracture or a pathological fracture.

In all patients, the trauma mechanism was analyzed and categorized into traffic accidents (car, motorcycle, bicycle or pedestrian), sports accidents, fall from height (>3 meters), and other (e.g. fall <3 meters).

Thoracic or clavicle x-ray imaging was performed in all patients, and additional computed tomography (CT) was performed when indicated. These imaging studies were used for fracture classification. Fractures were classified according to the Robinson classification [6]. The primary classification was anatomically divided into medial (Type I), middle (Type II) and lateral (Type III) thirds. Each of these types was then subdivided based on the magnitude of fracture fragment displacement. Fracture displacement less than 100% characterized subgroup A and displacement more than 100% characterized subgroup B. Fractures were classified by two researchers (JvL and SF). When no consensus was achieved, a third researcher’s vote (MH) was decisive.

To calculate the injury severity score (ISS), all injuries were allocated to one of six body regions [head and neck, face, chest, abdomen, extremities and external] and coded using Abbreviated Injury Scale (AIS) location codes [10].

Patients were divided in two groups based on the ISS: monotrauma and polytrauma patients. Monotrauma patients sustained a clavicle fracture or a clavicle fracture plus a minor abrasion, hematoma or superficial skin lesion leading to an ISS of 4 or 5 respectively. Patients were considered polytrauma when the ISS was ≥16 as a result of injury in 2 or more AIS regions [11]. Patients with a clavicle fracture and additional extremity fractures or cerebral contusion resulting in an ISS of 4 or 5 were excluded from analysis, as were all patients with an ISS of 6 to 15.

### Data analysis

Mean numbers were reported with standard deviation (SD). Statistical analysis was performed using the χ^2^ test for categorical variables and t-test and for continuous variables. Post-hoc analysis was performed using binary logistic regression with backward likelihood ratio. A p-value of ≤0.05 was considered significant. Data were analysed with SPSS Version 20.0, Chicago, IL, USA.

## Results

**Figure 1:**
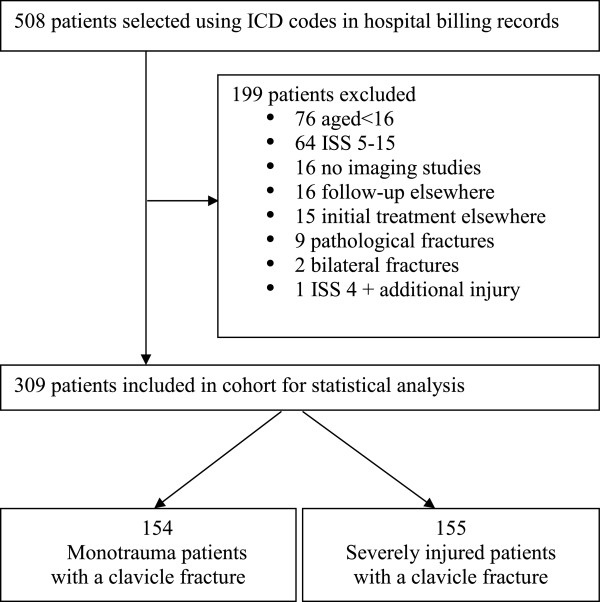
**Flow chart showing patient selection and exclusion.** 508 patients are initially selected and 199 patients are excluded from the analysis. Of the 309 patients included for the analysis 154 are monotrauma patients with a clavicle fracture and 155 are polytrauma patients with a clavicle fracture.

From 2007 – 2011, a total of 508 patients with a clavicle fracture were identified using the designated ICD code. Figure 1 shows the number of excluded patients and final group size. Table 1 shows demographics of all included patients with a clavicle fracture. There was no difference in affected side. In the primary survey, 85% of clavicle fractures were diagnosed by thoracic x-ray, and the remaining 15% were diagnosed by CT scan within 24 hours of admission.

**Table 1:** **Baseline characteristics of the studied population**

		Monotrauma N = 154		Polytrauma N = 155		P value
Age overall years (SD)		37.3	(17.1)	47.7	(20.8)	<0.001
Gender Male (%)		118	(76.6%)	106	(68.4%)	N.S.
HET* (%)		36	(23.4%)	110	(71.0%)	<0.001
ISS** (SD)		4.1	(0.24)	29.2	(10.2)	<0.001
Mortality during admission (%)		0	(0%)	32	(20.6%)	<0.001
Admission department	Surgery ward	37	(24.0%)	28	(18.1%)	N.S.
	Intensive care	0	(0%)	61	(39.4%)	<0.001
	Medium care	0	(0%)	41	(26.5%)	<0.001
	Operation room	1	(0.6%)	20	(12.9%)	<0.001
	Not admitted	116	(75.3%)	0	(0%)	<0.001
	Unknown	0	(0%)	5	(3.2%)	<0.001
Trauma mechanism	Traffic	58	(37.7%)	100	(64.5%)	<0.001
	Fall from height	1	(0.6%)	46	(29.7%)	<0.001
	Sports	61	(39.6%)	3	(1.9%)	<0.001
	Other	34	(22.1%)	6	(3.9%)	<0.001
Concomitant fractures of thorax and upper extremity	Costae			96	(61.9%)	N.A.
	Scapula			23	(14.8%)	N.A.
	Sternum			20	(12.9%)	N.A.
	Humerus			9	(5.8%)	N.A.

*HET; Patients involved in a high energy trauma **ISS; Injury of Severity Score, Mean noted with ±, standard deviation, N.S.; not significant, N.A.; not applicable.

Monotrauma patients had a higher incidence of Robinson type IIB (displaced) fractures compared to polytrauma patients, 74.5% versus 53.8% respectively (P = 0.002). There was no difference in Robinson type I and type III fracture distribution between monotrauma and polytrauma patients. Robinson type IIB fractures were treated operatively more often in monotrauma patients (P = 0.004). There was no difference in initial treatment choice for Robinson type I and III fractures between monotrauma and polytrauma patients. Treatment of Robinson type IIA and IIIA was conservative in all patients (Table 2). All 61 polytrauma patients admitted to the intensive care unit were treated conservatively.

**Table 2:** **Distribution of fracture types and treatment in monotrauma and polytrauma patients with a clavicle fracture**

Fracture type	N per type	Treatment	Monotrauma N = 154		Polytrauma N = 155		P value
**All patients**		Operative	26	(16.9%)	6	(3.9%)	**<0.001**
		Conservative	128	(83.1%)	149	(96.1%)	
**I** **-A**	**8**	Operative	0	(0%)	1	(14.3%)	N.S.
		Conservative	1	(100%)	6	(85.7%)	
**I-** **B**	**2**	Operative	0	(0.0%)	0	(0.0%)	N.S.
		Conservative	0	(0.0%)	2	(100%)	
**II** **-A**	**76**	Operative	0	(0.0%)	0	(0.0%)	N.S.
		Conservative	27	(100%)	49	(100%)	
**II** **-B**	**136**	Operative	19	(24.1%)	3	(5.3%)	**0.004**
		Conservative	60	(75.9%)	54	(94.7%)	
**III** **-A**	**68**	Operative	0	(0.0%)	0	(0.0%)	N.S.
		Conservative	38	(100%)	30	(100%)	
**III** **-B**	**19**	Operative	4	(44.4%)	1	(10.0%)	N.S.
		Conservative	5	(55.6%)	9	(90.0%)	

I; Robinson type I, II; Robinson type II, III; Robinson type III, A; not displaced, B; displaced, N.S.; not significant.

Multivariate analysis using binary logistic regression on patients with a DMCF showed that polytrauma patients received operative intervention 5.9 times less often than monotrauma patients, while accounting for age, gender, high energy trauma (HET) and trauma mechanism (P = 0.007; 95% CI 1.6-21.1). None of the other included variables were significant in the analysis.

## Discussion

Monotrauma patients had a higher incidence of displaced midshaft clavicle fractures (DMCF) compared to polytrauma patients and monotrauma patients with DMCF were treated operatively more frequently. No differences were found in the distribution and treatment of medial and lateral clavicle fractures.

In this study, polytrauma patients showed a lower incidence of DMCF (53.8%) compared to monotrauma patients (74.5%). This could be due to a difference in trauma mechanism in both groups. A clavicle fracture resulting from a solitary injury is most often caused by direct forces to the clavicle, e.g. direct blow to the shoulder in sports [12]. We assume that in polytrauma patients, forces are distributed over a larger area of the body, leading to rib fractures and/or pulmonary contusion. This assumption is supported by the relatively high rate of other fractures of the thorax and upper extremity (Table 1). A higher rate of sedation and intubation in polytrauma patients could also play a role as this may result in less traction of muscles on the fractured clavicle parts, thereby leading to less dislocation. In addition, polytrauma patients are more often imaged in the supine position during resuscitation at the emergency department, whereas monotrauma patients are more likely to be imaged in an upright position. The weight of the arm could therefore maintain fracture displacement.

In polytrauma patients, a clavicle fracture is not the primary concern and therefore treatment could be delayed. The high rate of additional injuries and intensive care admissions indicates the high level of care that conservatively treated polytrauma patients require (Table 1). Data from this study and other publications support high incidences of chest injuries in polytrauma patients [13, 14]. Chest injuries and compromised lung parameters may withhold the surgeon from starting operative treatment.

A large epidemiologic analysis of 25,000 polytrauma patients in Germany reported a rate of 60% of accompanying severe head and thoracic injuries (AIS score >2) in patients with a clavicle fracture [15]. These patients often have long periods of immobilization during admission or intensive care unit stay and postponed, slow rehabilitation [16, 17]. In patients with a traumatic brain or head injury, there is evidence of accelerated fracture union [18]. Both hyperventilation, to reduce intracranial pressure in traumatic brain or head injury patients, and a mild alkaline systemic environment are posed as hypotheses for this effect. Surgeons should be aware of this principle in polytrauma patients since it could alter the intervention options as well as the time window compared to monotrauma patients.

A recent meta-analysis of randomized clinical trials showed that the advantage of operative intervention in patients with a DMCF is predominately an early effect with no difference in function at one or two years follow-up [4]. Thus, the advantages of earlier return to normal function are obvious for an active adult population, however this might not be the case for polytrauma patients. Furthermore, operative intervention is not without risk. Both elastic stable intramedullary nailing and plate fixation are interventions with risks of complications [19, 20].

The debate about the optimal treatment for lateral displaced clavicle fractures (LDCF) does not differ much from that of DMCF. Higher numbers of non-union for conservatively treated patients and good functional outcome in operatively treated patients serve as arguments for surgical intervention [5, 21–23]. A recent meta-analysis on LDCF reported good results for operatively treated patients with respect to union. [24]. However, operative intervention for LDCF also has the risk of complications, and there is a high rate of implant revision and removal in these patients [23]. It is debated that although conservative treatment more often results in non-union, this does not always lead to a worse functional outcome. Thus, non-operative intervention should always be considered in polytrauma patients, and a wait-and-see approach in polytrauma patients with a DMCF or a DLCF is reasonable [23]. However, follow-up studies of polytrauma patients with DMCF or LDCF are lacking.

This study is limited by its retrospective nature and the lack of long-term outcome data in both groups. However, data derived from the Dutch National Trauma Database were prospectively collected, and the data’s accuracy is constantly evaluated by two database managers and a trauma surgeon. All the data were collected in one level 1 trauma center thus excluding institution preference influence. In addition, using thoracic x-rays to determine the Robinson classification can be difficult, especially with respect to sub-classification. Therefore, only the most essential elements of the classification, fracture location (medial, midshaft or lateral) and displacement, were used in this study. Another drawback is that the indication for operative treatment was not provided in this study. In our hospital, absolute indications for operative treatment of midshaft clavicle fractures include open fractures or fractures where the overlying skin is imminently threatened, neurovascular injury, and floating shoulder. A relative indication for operative treatment of midshaft and lateral fractures is displacement (Robinson type B fractures).

This is the first study comparing the distribution and treatment in monotrauma and polytrauma patients with a clavicle fracture. The results indicate that monotrauma and polytrauma patients with a clavicle fracture are different entities. It incites further research on the functional outcome, patient satisfaction, and complications of operative and conservative treatment in polytrauma patients with a clavicle fracture.

## Conclusions

Monotrauma patients had a higher incidence of displaced midshaft clavicle fractures (DMCF) compared to polytrauma patients and monotrauma patients with DMCF were treated operatively more frequently. No differences were found in the distribution and treatment of medial and lateral clavicle fractures.
